# Editorial: The roles of self-organization and sensory adaptation for locomotion in animals and robots

**DOI:** 10.3389/fnbot.2024.1372772

**Published:** 2024-02-06

**Authors:** Bulcsú Sándor, Claudius Gros, Poramate Manoonpong

**Affiliations:** ^1^Department of Physics, Babes-Bolyai University, Cluj-Napoca, Romania; ^2^Institute for Theoretical Physics, Goethe University Frankfurt, Frankfurt am Main, Germany; ^3^Bio-inspired Robotics and Neural Engineering Lab, School of Information Science and Technology, Vidyasirimedhi Institute of Science and Technology, Rayong, Thailand; ^4^Embodied Artificial Intelligence and Neurorobotics Lab, SDU Biorobotics, The Mærsk Mc-Kinney Møller Institute, University of Southern Denmark, Odense, Denmark

**Keywords:** robotic locomotion, motion patterns, swarms of robots, control and learning algorithms, sensory feedback loop

## 1 Introduction

Self-organization is the emergence of a global spatial or dynamical pattern in a system with interacting components governed by simple local rules (Gros, 2015). In the context of animal and robotic locomotion, self-organization may manifest itself on two complementary levels. On one hand, the motion of individual agents may emerge in a self-organized manner as a result of the dynamical interaction of the controlling brain and the physical body of the robot, which in turn is in contact with its surrounding environment (Pfeifer et al., 2007; Der and Martius, 2012; Sándor et al., 2015; Thandiackal et al., 2021). On the other hand, for systems with multiple agents, the interaction between the robots or animals may allow in some cases to solve tasks seemingly impossible for the individuals (Rubenstein et al., 2014). For both levels, sensory adaptation provides a way for brain-body, body-environment, and agent-agent interactions. The goal of this Research Topic is to consolidate results related to “*The roles of self-organization and sensory adaptation for locomotion in animals and robots*,” and to contribute to our understanding of self-organizing principles for locomotion. The present Research Topic contains six articles, addressing strategies of self-organized locomotion for legged and wheeled robots on the level of single agents and for swarming.

## 2 Self-organized locomotion

Attaining robust, self-organized locomotion like animals is a crucial feature for legged robots. This bio-inspired strategy enables robots to autonomously form their gaits within seconds through local feedback loops and dynamic body-environment interactions, without requiring robot kinematics models, environmental models, predefined interlimb coordination, or a large amount of training data (Aoi et al., 2017; Sun et al., 2021).

An important local feedback loop for the locomotion of insects is provided by strain sensing in parts of the leg that are subjected to high loads. To better understand the role of the load feedback signals, Zyhowski et al. developed a robotic leg that incorporates mechanical strain gauges emulating their biological counterpart sensors, while their output is processed by a dynamic computational model of the receptors. Performing a series of experiments, they found that this sensory setup is able to reliably signal increasing and decreasing force scenarios at the beginning and at the end of the stance phase, respectively, in different stepping conditions. Such sensory adaptation may provide a crucial contribution to the emergent intra- and inter-leg coordination during locomotion on complex terrain.

Following the bio-inspired control strategy, Sun et al. proposed adaptive neural control with adaptive physical and neural communications (APNC). The control integrates distributed local central pattern generator (CPG)-based neural circuits for generating basic leg movements, an adaptive sensory feedback mechanism for generating self-organized phase relationships among the local CPG circuits, and an adaptive neural coupling mechanism for transferring and storing the formed phase relationships (a gait pattern) into the neural structure. The control approach can generate robust self-organized locomotion of quadruped robots under various situations (including sensor malfunction, uneven terrain, noisy feedback, leg damage, carrying a payload, different locomotion speeds, and different control update frequencies). The work also provides a better understanding of the roles of self-organization and sensory adaptation for generating adaptive, robust, and reusable locomotion.

In addition to the self-organized locomotion and sensory adaptation of quadruped robots, Mingchinda et al. investigated various strategies of coordination between multiple legs and body segments of multi-segmented, multi-legged robots with more than six legs during turning for navigation in complex environments (e.g., narrow spaces). The investigated leg and body coordination strategies include (i) the local leg and body coordination at the segment level (LCS) in a manner similar to millipedes, simultaneous leg amplitude reduction in response to different turning directions (SAR, like insects), and the global phase reversal of legs inside of turning curve (GPR, typical engineering approach). The results show that among all control strategies LCS and GPR performed equally well and outperformed SAR for the narrow square maze. In the more complex zigzag maze, where turning in two directions is necessary to escape the environment, GPR demonstrated the highest performance in terms of locomotion speed within narrow spaces. In a nutshell, the study illustrates that various leg-body coordination strategies exhibit distinct preferences in different situations, providing valuable insights for guiding the development of controls for complex, multi-segmented, legged robots.

Valencia Urbina et al. investigated the behavior of two-wheeled robots in a Braitenberg-style obstacle avoidance setup, but with a controller based on the connectome of *C. elegans*. In a general context, two-wheeled robots have been used for a wide range of purposes, such as the emergence of kick control (Sándor et al., 2018). Here, Valencia Urbina et al. showed, that it is possible to transfer a biological network of neurons to a functioning robotic application. While the neurons of the controller are identical in terms of their firing threshold, coexisting clusters of oscillators with distinct characteristic frequencies emerge due to the structure of the *C. elegans*' connectome. The stimulation of sensory neurons promotes the emergence of obstacle avoidance ability, making the robot move backward solely due to the activation of a small number of units.

In a separate study by Guerrero-Criollo et al., biologically inspired controlling circuits for robots with two wheels were investigated, this time in the framework of spiking neural networks. Putting together short-term memory circuits with winner-take-all and modulation neural networks, the authors showed for maze navigation tasks, that the performance achieved using biologically inspired networks is comparable to that of dedicated architectures.

Self-organizing processes may lead to group-level intelligence through the communication of multiple interacting agents allowing for solving complex problems despite being composed of relatively simple units. In their review paper, Wang et al. survey different swarm intelligence algorithms and setups developed for odor source localization (OSL). In the OSL task, single or multiple agents search, discover, and identify chemical plumes in their environment by using their sensors. Swarm intelligence algorithms have the advantage that multiple robots together can localize the odor source more robustly when odor diffusion is perturbed by turbulence and avoid trapping into locally optimal solutions. While Wang et al. mostly focus on variants of particle swarm optimization algorithms, various bio-inspired heuristic optimization methods are also discussed together with the active challenges of robot olfaction research.

## 3 Concluding remarks

The present Research Topic brings together studies focusing on different aspects of self-organized locomotion starting from the role of load feedback signals, through bio-inspired control strategies for intra- and inter-leg coordination, biologically realistic neural networks for obstacle avoidance and maze navigation tasks, to odor source localization by swarms. As demonstrated by these works, using self-organizing processes may reduce the computational complexity of the algorithms designed to solve several real-world-inspired tasks while being also robust to sensory noise and environmental perturbations.

## Author contributions

BS: Writing—original draft, Writing—review & editing. CG: Writing—original draft, Writing—review & editing. PM: Writing—original draft, Writing—review & editing.
